# From Drops to Decisions: AI/ML-Driven Biofluidics for Clinical Diagnostics and Healthcare Intelligence

**DOI:** 10.1021/acs.analchem.5c06356

**Published:** 2025-12-23

**Authors:** Mayuri Tushar Deshmukh, Yogesh Thakre, Wani H. Bisen, K. Sankar, Ankita Avthankar, Aniket K. Shahade, Manish Bhaiyya, Madhusudan B. Kulkarni

**Affiliations:** † Department of Electronics and Telecommunication Engineering, 226452 Marathwada Mitra Mandal’s College of Engineering, Pune, MH 411052, India; ‡ School of Computer Science and Engineering, Ramdeobaba University, Nagpur 440 013, India; § Department of Computer Science and Engineering, G H Raisoni College of Engineering, Nagpur, MH 441110, India; ∥ Department of Artificial Intelligence and Data Science, 306438 Vel Tech High Tech Dr Rangarajan Dr Sakunthala Engineering College, Avadi, Chennai, TN 600062, India; ⊥ Symbiosis Institute of Technology,, 29630 Symbiosis International (Deemed University), Nagpur Campus, Pune, MH 440035, India; # Symbiosis Institute of Technology, 29630 Symbiosis International (Deemed University), Pune Campus, Pune, MH 412115, India; ∇ EQIQAI Systems Private Limited, Kokapet, Rajendra Nagar, K. V. Rangareddy, Hyderabad, TG 500075, India; ○ Department of Electronics and Communication Engineering, Manipal Institute of Technology, 76793 Manipal Academy of Higher Education (MAHE), Manipal, KA 576104, India

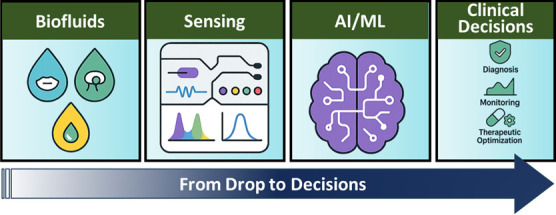

## 1 Introduction

Biofluidics couples the microfluidic handling of minute quantities of body fluids, namely blood, saliva, urine, sweat, and tears, with on-chip sensing methodologies such as spectroscopy, electrochemical signals, color changes, and imaging.^1–4^ This allows the retrieval of crucial biochemical information in a timely manner and close to the patient. By shrinking steps such as sample preparation, reactions, separation, and detection into the microlitre or even nanolitre scale, biofluidic systems use fewer chemicals and finish tests faster, while easily connecting to portable readers such as smartphones or small edge devices. In other words, biofluidics moves the complete testing format out of large laboratories onto small, compact devices that are able to provide results from sample to answer.^5–9^ We face manifold challenges in our health systems today: aging populations, chronic disease, antibiotic resistance, and the rise of remote or telehealth. We need diagnostic tools that are cheap, usable often, and that can be used outside of the big hospital laboratories.^10–13^ Biofluidics can be the key technology for this change. It allows easy, friendly and minimally invasive sample collection, enables repeated testing, and works in point-of-care, at home, small clinics, ambulances, or low-resource settings. More than just one-off tests, biofluidics wearables and connected devices transmit continuous or frequent readings that provide long-term captures of the patient’s real physiology, treatment, or decline, and early warning of worrisome changes.^14–16^ Everyday hardware is used; LEDs, smartphone cameras and low-power electrochemical readers, for example, combine with scalable manufacturing approaches (roll-to-roll paper devices, injection-molded cartridges, screen-printed electrodes) to democratize these technologies to make them cheap and fast to adopt.^17,18^

Centralized assays, while highly sensitive and specific, often require venous blood draws, cold-chain logistics, skilled operators, and batched workflows with turn-around times measured in hours to days.^19–21^ Costs are dominated by labor and infrastructure; access is limited for rural and low-income settings; and results typically represent a single snapshot in time, missing temporal dynamics critical for conditions such as sepsis, heart failure decompensation, glycemic control, or therapeutic drug monitoring.^22,23^ Moreover, translating complex spectra or electrochemical signatures into clinically meaningful numbers usually demands expert interpretation, creating bottlenecks and variability.^24^ AI closes these gaps by turning raw, noisy readouts into calibrated, actionable outputs at the edge. Classical chemometrics, ML classifiers, and modern deep learning enable robust quantification and classification directly from Raman, electrochemical curves (CV/DPV/EIS), colorimetric bands, or microfluidic images.^3–26^ AI also supports personalization (federated/continual learning, domain adaptation for lot-to-lot and site-to-site shift), trust (uncertainty estimation, calibration, explainability), and operations (quality control, anomaly detection, device self-diagnostics).^27,28^ In connected ecosystems, models transform repeated biofluid measurements into risk trajectories and decision support, for example, dose titration in therapeutic drug monitoring, infection triage from saliva/urine, or cancer screening via volatile or spectral biomarkers.^29,30^

Several prior reviews separately survey microfluidic technologies, wearable chemistry, or AI for digital health. By contrast, this review is organized around clinical decision points rather than devices alone. While numerous reviews have independently addressed microfluidic device engineering, wearable chemical sensors, or AI applications in digital health, they largely remain siloed, focusing on either materials, fabrication, or algorithmic performance in isolation. Recent surveys in Dang-Khoa Vo, Zhou SK, Kimia Zarean Mousaabadi, Kuldeep Mahato, and Andrei Bocan predominantly highlight device miniaturization, multiplexed assays, or ML-based data analytics, but rarely connect these domains through the lens of clinical decision-making.^31–35^ Other thematic reviews (e.g., on AI in biosensing, wearable diagnostics, federated learning in healthcare, and biofluid-based nano plasmonic sensing) often emphasize technical innovation without mapping how such tools translate into actionable clinical end points, validation frameworks, or regulatory readiness.^15,36,37^ Conversely, this review attempts to pull these vignettes into a starting roadmap centring on decisions and diagnosis as an end-goal, linking biofluid type, analytical readout, and AI-driven inference to the critical clinical steps of diagnosis, monitoring, and therapeutic optimization. Rather than classifying technologies on the basis of sensor or algorithm class, we focus on how analytical performance, model calibration, and decision-curve utility all coalesce to establish translational credibility. This framework, visualized schematically in Figure 1, attempts to span the chasm between the benchtop of sensing innovation and the bedside of clinical reasoning, aspiring to inspire research and clinical practice from drops of biofluid to defensible clinical action.

**Figure 1:**
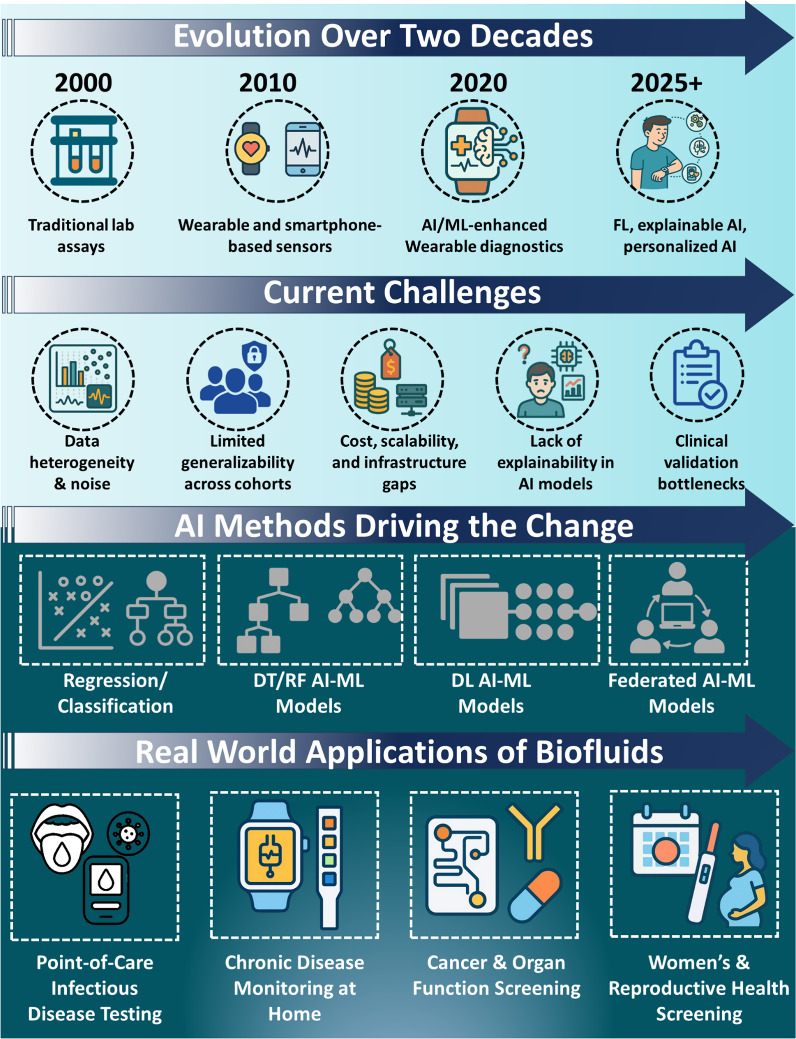
Overview of AI-enabled biofluidics: Timeline from lab assays to explainable/federated AI, key challenges, the ML methods driving progress, and real-world applications.

It is combined to provide logical development from analytical underpinnings to translational outcomes. Section 2 introduces the analytical and engineering underpinnings of biofluidic devices, concentrating on microfluidic integration, sensing modalities, and mechanisms of emitting signals. Section 3 describes the spectrum of AI/ML models in play from classical chemometrics to DL and federated algorithms, focusing on their roles in feature extraction, quantification, and inference. Section 4 describes clinical applications to all major biofluids-saliva, sweat, urine, and tears-that highlight how AI/ML-mediated biofluidics target infectious, metabolic, oncologic, and reproductive health. Section 5 consolidates the important evaluation metrics and performance hierarchies for analytical validity, algorithmic reliability, and clinical impact. Section 6 provides a critical examination of deployment-related challenges, including data quality, domain shift, security, and regulations, translated it into action-oriented priorities for near-term and longer-term objectives for stakeholders in research, clinical practice, and policy. Finally, the Conclusions and Future Directions section reflects on the next decade of AI-enabled biofluidics and highlights a plan of action from laboratory innovation to patient-centered diagnostics that can scale globally.

## 2 AI–ML Models at the Heart of Biofluid Intelligence

“Pure” biochemical data gleaned from biofluids can only be as useful as the intelligence of the models that read them. After microfluidic and biosensing devices have been used to detect an optical, electrochemical, or colorimetric signal, how is this signal decoded into the next biochemical or physiological message? AI is the translator in this chain, taking spectral peaks, voltage transients, and pixel intensities and giving them probabilistic prediction and diagnostic meaning. This progression has been linear over the last 20 years: from classical chemometric regressions generating calibration curves, to data-driven ML classifiers learning diagnostic margins, to DL architectures capable of end-to-end learning and multimodal reasoning. These approaches collectively represent the computational backbone of modern biofluidics, the link from microscopic drops of information to megascopic clinical meaning.^38^

## 3 Learning from Linearity: Classical AI in Biofluidics

Before deep neural networks became prevalent, biofluidic data were interpreted using mathematical and rule-based models. They laid the essential foundation for associating spectral or electrochemical signals to real-world biochemical concentrations. Even if the models are constrained since they assume linearity, they contain the earliest examples of algorithms modeling human decisions on measurement, identification and validation of molecular information.

### 3.1 Chemometric Regression Models

Traditional biofluidic calibration commonly employs Partial Least Squares Regression (PLSR), Principal Component Regression (PCR), and Multiple Linear Regression (MLR), see Figure 2A. These models reduce multicollinear spectral or electrochemical features into latent variables that best correlate with reference analyte concentrations.^39,40^ They are especially suited for quantitative assays such as glucose or lactate estimation from Raman, FTIR, or impedance spectra. Their simplicity, transparency, and low computational cost make them ideal for embedded use in portable readers.^41,42^ However, they assume linearity and can degrade in nonlinear regimes or when strong matrix effects distort signal proportionality.^43,44^

**Figure 2:**
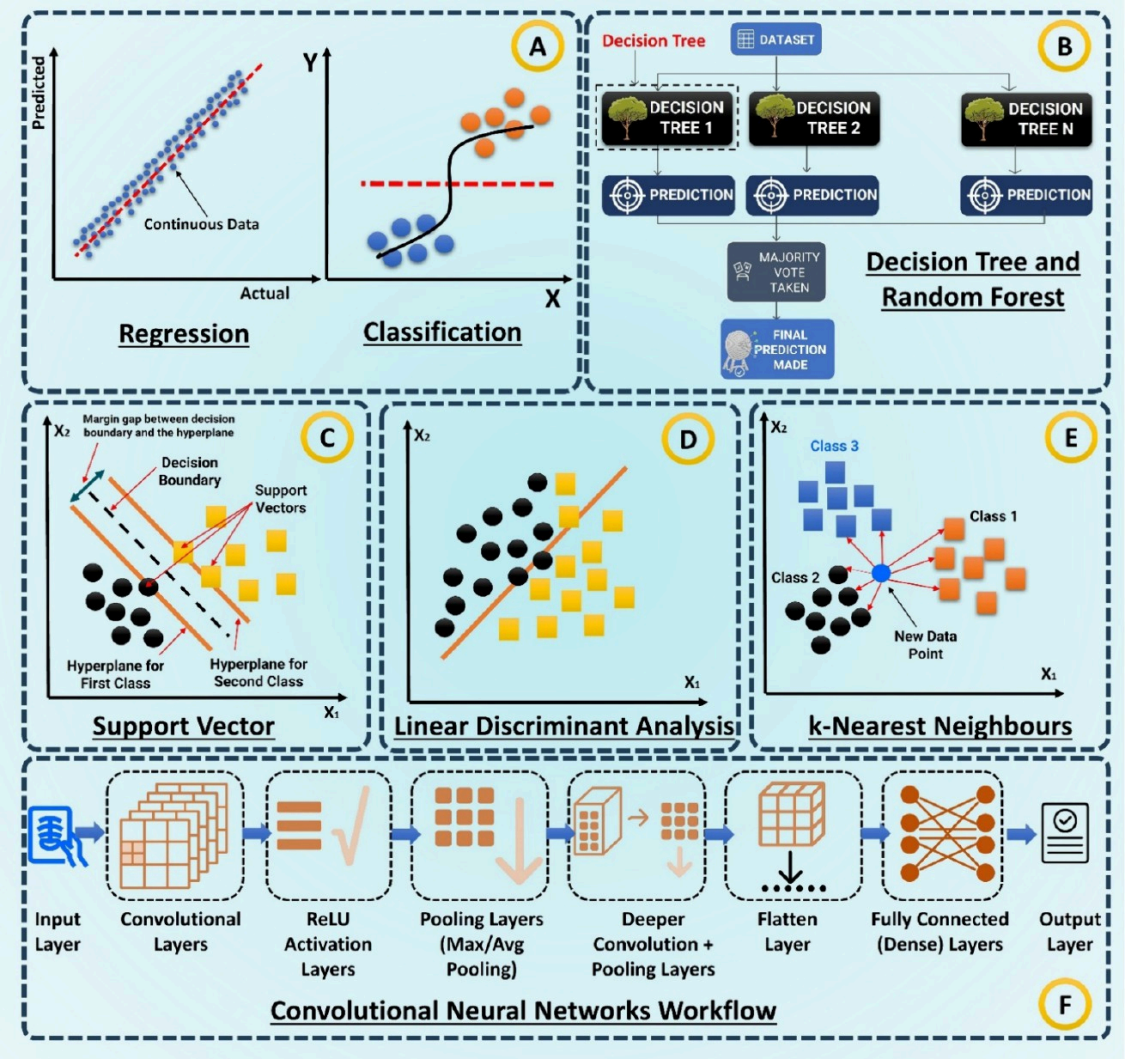
Core ML models used in biofluid analytics. (A) Regression vs classification. (B) Decision tree/Random forest voting. (C) SVM margin. (D) LDA linear boundary. (E) k-NN proximity. (F) CNN pipeline from convolution/pooling to dense output.

### 3.2 Classification and Discriminant Algorithms

When the task involves disease/no-disease or class differentiation, Support Vector Machines (SVM), Linear Discriminant Analysis (LDA), *k*-Nearest Neighbors (*k*-NN), Decision Trees, and Random Forest (RF) are frequently applied, see Figure 2B–E.^45,46^ These algorithms partition feature space into interpretable boundaries and provide probability-based outputs suitable for diagnostic classification, for example, saliva-based infectious-disease triage or urine-based renal-function stratification.^47,48^ Because they train effectively on limited labeled data and tolerate moderate noise, they remain essential in early stage prototypes and regulatory submissions where explainability is mandatory.

### 3.3 Feature Selection and Dimensionality Reduction

Principal Component Analysis (PCA), Independent Component Analysis (ICA), and Recursive Feature Elimination (RFE) help suppress background variation and highlight informative variables.^49,50^ Applied before model fitting, these steps improve stability, reduce overfitting, and enable real-time computation on low-power microcontrollers or smartphones.^51^ Nevertheless, shallow models depend heavily on preprocessing, baseline correction, normalization, detrending, and struggle with nonlinear signal–response relationships typical of multianalyte biofluids.^43^

## 4 Deep-Learning Architectures

While classical algorithms would provide the first, basic skeletal framework for biofluid data analysis, they were tied to the tower of hand-crafted features as well as simplistic, linear assumptions on how those predictors mapped to the final diagnosis: they could only go so far. Furthermore, as sensors became more and more sophisticated, throwing out full spectra, voltammograms, image streams, etc., we started to need models that learnt directly from raw data: this was the rise of deep learning - neural nets finding hidden nuggets we never could.

### 4.1 Convolutional Neural Networks (CNNs)

CNNs dominate image-based and colorimetric biofluidics. They automatically learn hierarchical spatial features such as color gradients, fluorescence zones, or microchannel patterns, shown in Figure 2F.^52,53^ By mitigating dependence on manual feature engineering, CNNs achieve robust quantification under varying illumination or camera angles, enabling smartphone-based lateral-flow immunoassay analysis and on-chip colony detection.^54^

### 4.2 Temporal and Sequential Models

In the case of electrochemical signals or impedance-overtime data, models such as CNNs, RNNs, and LSTMs can learn temporal variations in the signal. They are able to detect small peaks of current or slow relaxation patterns that correspond to biochemical reactions. This makes them useful in the real-time monitoring of metabolites, such as glucose levels in sweat, or measuring antibiotic levels in drug-monitoring systems.^55,56^

### 4.3 Unsupervised and Generative Frameworks

Autoencoders (AEs) and Variational Autoencoders (VAEs) build compact, noise-robust latent spaces from unlabeled spectra or images. These representations support device-to-device calibration transfer, anomaly detection, and domain adaptation arising from changes in sensor lots or assay chemistries. By retaining the essential noise-free variance inherent to the data while filtering out measurement noise, they improve reproducibility without costly, extensive retraining.^57,58^

### 4.4 Multimodal and Transformer Models

Recently developed Transformer-based architectures that fuse heterogeneous inputs (spectral, electrochemical, and optical), as well as contextual metadata (age, medication, environment) into a single latent representation. Self-attention allows the model to focus on the most informative locations or time points to boost robustness against heterogeneous or incomplete biofluid data, which is in line with the field’s movement toward integrated digital-twin-style where minor modalities inform clinical decision making.^59,60^

### 4.5 Explainability and Interpretability Overlays

It is important that rationales are cohesive with regulatory and clinical usage. Visualization approaches such as Grad-CAM, SHAP, and Integrated Gradients highlight which bands of spectra or patches of images contributed to a particular prediction. Those visualization approaches, combined with calibration scores that integrate the Brier score and ECE, along with out-of-distribution detection, give deep networks trustworthy powers and make them auditable diagnostic aids that account for the gap separating black-box AI from clinical accountability.^61,62^ Put together, classical and DL frameworks therefore represent the complementary pillars of modern biofluid analytics, whereby one offers interpretability and calibration precision, while the other enables autonomous pattern discovery across diverse signal types. Finally, the true value of any model resides not in its architecture, but in the reliability of its performance under real clinical conditions. Therefore, the next section of this writeup will provide a discussion of the key analytical, computational, and clinical metrics that can be used to quantify this performance and measure when AI solutions in biofluidics can be trusted.

## 5 Key Metrics Used (Analytical, ML, and Clinical Impact)

The assessment of the performance of AI-biofluidic systems necessarily requires a multidimensional perspective, from analytical fidelity at the sensor level, through algorithmic fidelity at the model level, to, ultimately, clinical or economic value at the point of decision. Metrics are the common currency of the engineer, the data scientist, the clinician: they provide a vocabulary for how well a signal, a model or a whole diagnostic pipeline performs in practice. In some cases, performance is clear-cut. In others, especially those in which learnings emerge from ongoing deployments, one can use a combination of the indicators presented below as a single analogy, it is not just the scoreboard but the process that matters. This section outlines some of the most significant features categorized under analytical performance and ML performance that, in sum, provide the foundation for believable, comparable and clinically useful performance.

## 6 Analytical Performance

Before implementing any AI layer to create value, the proposed assay must have a firm foundation of analytic validity. These are metrics that define the extent to which a biofluidic device relates molecular information to measurement signals with correctness and precision.

### 6.1 Sensitivity, Specificity, and Detection Limits

The Limit of Detection (LoD) and Limit of Quantification (LoQ) denote the concentration of analyte, which can realistically be detected and quantified. These values ascertain if an assay using biofluidics can capture physiological changes, early infection biomarkers, or metabolites that may lie at low levels.^63–65^ The term linear range indicates the concentration range in which signal intensity remains closely tied to analyte concentration and, thus, is suitable for quantitative use. Sensitivity, on the other hand, is defined as the test’s ability to correctly identify all true positives, and specificity is defined as the ability to correctly identify true negatives, or reject false positives; both of which must be properly balanced in order to result in a viable diagnostic.^64,66,67^

### 6.2 Repeatability, Reproducibility, and Stability

Repeatability assesses performance under the same device and operator. Reproducibility assesses performance across batches of testing or across users or instruments and is expressed in the form of intraclass correlation coefficients (ICC). Stability against these conditions is also important for wearable or point-of-care systems, as this can affect the shelf life of the device and deployability to the field.

### 6.3 Cross-Reactivity and Matrix Effects

Typical biological fluids (saliva, urine, sweat, blood, etc.) often have interfering species, which may be proteins, ions, or metabolites, that alter true responses. Cross-reactivity explains the unwanted response attributed to nontarget interference, whereas matrix effects refer to the degree of signal alteration attributable to the physical–chemical characteristics of the biological fluid. Strategies for minimizing cross-reactivity and matrix effects are selective coatings, blocking agents, and computational correction models to ensure analytical accuracy in saliva, urine, sweat, or blood matrices.^44,68,69^

### 6.4 Operational Efficiency and Cost

The practicality of this assay is measured by metrics such as time-to-result, sample volume, and cost of consumables. Its fast turnaround time, sample consumption in the range of microliters, and inexpensive reagents make it feasible for use at the point-of-care or even within the home. Taken together, these three metrics establish the credible ground truth from which the AI algorithms can then have some trust in the result.^70,71^

## 7 ML Performance

Once the sensor output is confirmed, the next step is the ML layer, which serves as the brain that interprets the raw signals and returns a diagnosis. ML quality metrics fit into five categories that look at different aspects of model quality: discrimination, regression, calibration, robustness, and uncertainty.

### 7.1 Discrimination Metrics

These are measures of the effectiveness of a model in discriminating between positive and negative cases. The Area Under the Receiver Operating Characteristic curve (AUROC) provides a measurement of global discriminative power, while for imbalanced data sets, as is the case in rare diseases, the Area Under the Precision–Recall Curve (AUPRC) is preferred. Accuracy, F1-score, sensitivity, and specificity are global measurements to report a model’s performance within a threshold as complementary measurements.^18,72^

### 7.2 Regression Metrics

For continuous outcomes, such as analyte concentration or hormone level, Root-Mean-Square Error (RMSE), Mean Absolute Error (MAE), and Coefficient of Determination (*R* ^2^) measure the closeness of predicted and reference values.^65,73–75^

### 7.3 Calibration and Reliability

It is possible for a model to exhibit high levels of accuracy while misrepresenting the calibration of predictive probabilities. The Brier score is useful to quantify the similarity between the predicted probabilities and the actual outcomes. Calibration curves may also be employed for a visual evaluation of the correspondence. The concepts of Expected Calibration Error (ECE) and Maximum Calibration Error (MCE) effectively summarize the performance of a model across probability bins. Among more formalized eras of diagnostic regulation, calibration metrics are a primary means to ensure that predicted risk values represent the true likelihood of risk in clinical practice.^65,73,74^

### 7.4 Robustness and Domain Shift

Deployment in the real-world context exposes models to new sensors, users, or environments, which may degrade performance. Cross-device or cross-site AUC drop assesses performance drop when the model is used in new domains. Out-of-distribution (OOD) detection metrics quantify when incoming data seems to differ from the training distribution, which may trigger an alarm or recalibration of the model. Together, cross-domain performance and OOD detection metrics contribute to characterizing a system’s generalizability or robustness, which are key necessary conditions to scale biofluidic AI.^64,65,73,74^

### 7.5 Uncertainty and Explainability

In what way might we enhance credibility with clinicians? Predicting well is not enough; you also need to project confidence in your prediction. Negative log-likelihood (NLL) or prediction-interval coverage indicate how good your probabilistic outputs are at explaining the true outcome of interest. How do we check to see if our models are looking in the right places? Explanation checks on the attention maps provide an assessment to ensure the model is indeed looking where the signal is, not the noise.^76^ These “sanity checks” serve to ensure that decisions are informed by biological rather than spurious relationships. Lastly, Table 1 presents the layered performance hierarchy that integrates analytical, machine learning and clinical relevance within a single framework from sensor validation to algorithmic resilience of AI-enabled biofluidic systems.

**Table 1:** Key Metrics Used for Evaluating AI-Enabled Biofluidic Systems

**level**	**metric/parameter**	**what it measures**	**typical unit/method**	**why it matters (clinical relevance)**
analytical performance	LoD/LoQ	smallest detectable or quantifiable analyte concentration	μM, ng mL^–1^, or equivalent	detects trace biomarkers for early diagnosis
	linear range	proportionality between signal and analyte concentration	regression (*R* ^2^ ≥ 0.99)	ensures quantitative reliability
	sensitivity/specificity (assay)	true-positive and true-negative rates of sensor response	% or ratio	defines diagnostic accuracy and cross-reactivity control
	repeatability/reproducibility	consistency within and across runs, batches, or operators	CV (%), ICC	confirms analytical robustness and manufacturing quality
	stability	signal retention over time and environmental stress	ΔSignal %, shelf life (days)	predicts field durability and storage reliability
	cross-reactivity/matrix effects	interference from nontarget species or sample composition	relative error (%)	indicates selectivity and real-sample performance
	time-to-result	duration from sample to readout	minutes	critical for point-of-care turnaround
	sample volume	minimum sample required for valid detection	μL or nL	enables minimally invasive testing
	consumable cost	material and reagent expense per test	USD/test	determines affordability and scalability
ML performance	discrimination	Model’s ability to distinguish positive vs negative cases	AUROC, AUPRC, accuracy, F1	core diagnostic separability metric
	regression accuracy	quantitative prediction vs reference	RMSE, MAE, R^2^, Bland–Altman bias	validates quantitative agreement with gold standard
	calibration	agreement between predicted probability and reality	brier score, ECE, MCE	ensures probability outputs reflect true risk
	robustness/domain shift	performance drop under new device, site, or population	ΔAUC (%), OOD metrics	tests generalization and transferability
	uncertainty estimation	reliability of individual predictions	NLL, prediction intervals	flags low-confidence or ambiguous results
	explainability/sanity checks	model transparency and feature attribution	SHAP, Grad-CAM, saliency maps	Builds clinician trust and regulatory confidence

## 8 Translational Applications of AI-Integrated Biofluidics

Taking these mechanistic elements and informatic contexts, next, we convert them into diagnostic contexts via biofluids. Each of saliva, sweat, urine, and tears carries with it specific biochemical fingerprints and access features that make them ideal candidates for certain clinical situations. Coupled with AI/ML-based pattern recognition, biofluidic systems can turn raw sensor streams into accurate diagnostic insights. Below, we cover how biofluidics integrated with AI are being deployed for infectious disease screening, metabolism and stress monitoring, cancer and organ function diagnostics, and ocular/systemic disease detection; together, these case studies illustrate the intersection of analytical precision, ML intelligence, and clinical utility in bringing diagnostics from centralized laboratories to decentralized clinics at point-of-need for intelligent, real-time healthcare.

### 8.1 AI-Assisted Saliva-Based Diagnostics for Infectious and Metabolic Diseases

AI/ML-enabled saliva diagnostics are enabling speedier and more accessible disease detection in a noninvasive manner. Saliva, which is full of biomarkers such as proteins, antibodies, metabolites and nucleic acids, provides a painless and conveniently collected substitute for blood and tissue samples.^77,78^ Coupled with AI/ML algorithms, the sample’s complex spectral/imaging data can be accurately decoded into a disease prediction with no additional complex lab infrastructure required. This integration enables real-time monitoring, personalized risk assessment, and early diagnosis of infectious, metabolic, and oncological conditions.^14^

Inspired by this prospect, a variety of studies have recently demonstrated how saliva-based sensing, combined with AI/ML technology, is transcending categories to impact diagnostics. Liang et al. reported a smartphone-based paper microfluidic platform for quantifying tetrahydrocannabinol in human saliva using a fluorescence competitive immunoassay (Figure 3A). The sensor boasted a limit of detection of 1 pg/mL and was cross-reactant to cannabidiol, but quantification was enhanced to 88% with the use of ML SVM and k-NN, and 100% accuracy in classifying positives in the samples, demonstrating that AI-enhanced microfluidics holds promise for drug screening applications.^79^ Going beyond the mechanics of substance detection, Braz et al. adopted an e-tongue for the detection of salivary cancer markers. Using impedance spectroscopy combined with ML algorithms, distinguishing between saliva from oral cancer patients and healthy individuals was possible (see Figure 3B). Here, supervised models, mainly using SVM and random forest, achieved >80% accuracy for a binary classification task, and the integration of clinical parameters such as alcohol consumption further improved performance.^80^

**Figure 3:**
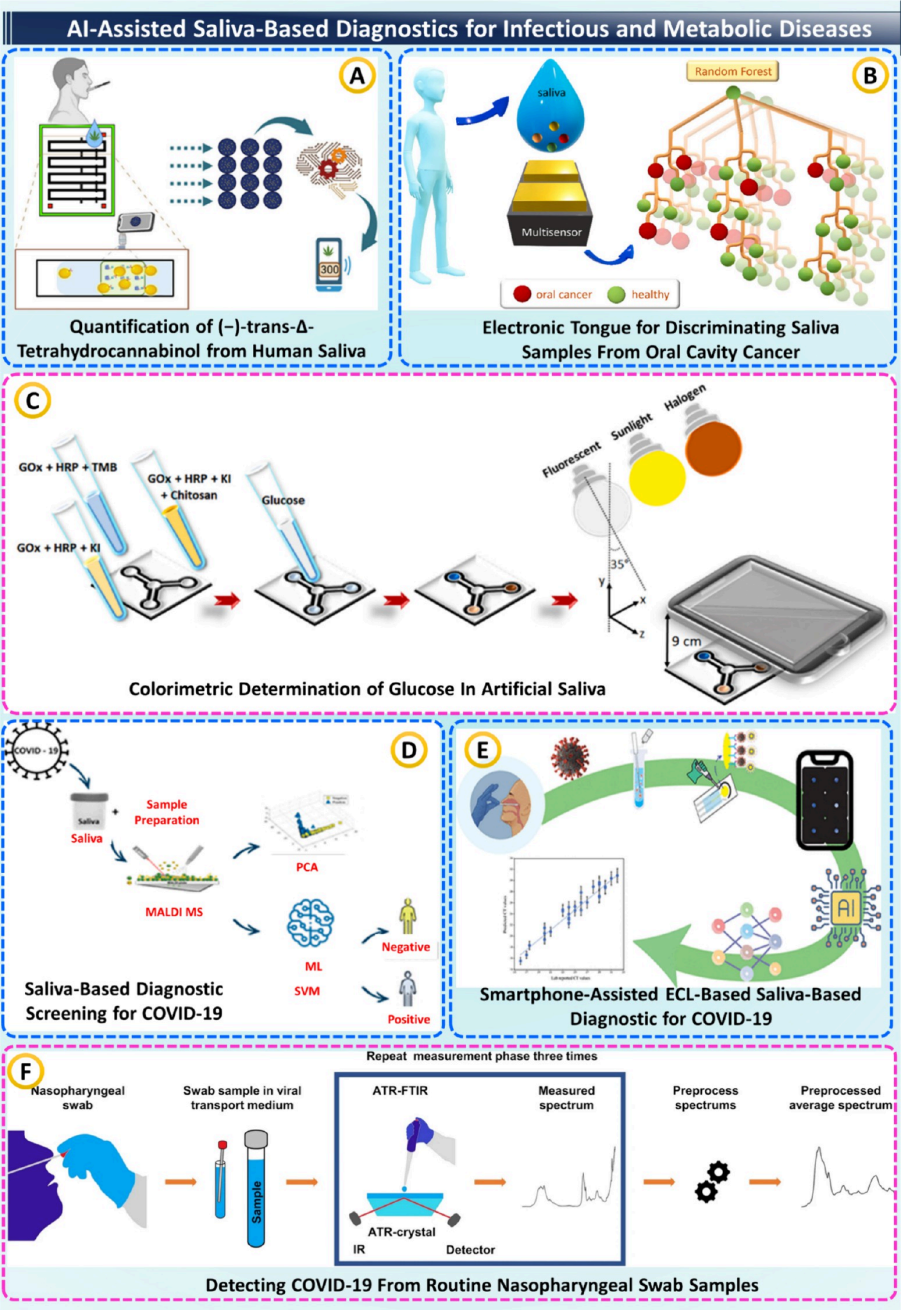
AI-assisted saliva (and swab) diagnostics across chemistries and ML models. (A) AI-aided quantification of (−)-trans-Δ-THC from human saliva, taken from^79^ with the permission of ACS. (B) Multisensor “electronic tongue” with RF separates oral-cancer vs healthy saliva, taken from^80^ with the permission of Elsevier. (C) Paper-microfluidic colorimetric glucose assay imaged by smartphone under varied illumination/angle, taken from^81^ with the permission of Elsevier. (D) Saliva MALDI-MS fingerprints + PCA/SVM for COVID-19 screening, taken from^82^ with the permission of ACS. (E) Smartphone-assisted ECL saliva test with ML regression/classification, taken from^84^ with the permission of ACS. (F) Reference pipeline: nasopharyngeal swab ATR-FTIR with repeat measurements and preprocessing for COVID-19 detection, taken from^85^ with the permission of Springer.

Shifting gears from disease detection to metabolic monitoring, Mercan et al. devised an ML-supported colorimetric μPAD for glucose quantification in artificial saliva. Users could capture images through a smartphone while using different lighting conditions (see Figure 3C). The entire system harnessed cloud-based ML classifiers, integrated within an Android app (“GlucoSensing”), yielding 98.24% accuracy along with excellent interdevice repeatability. An approach that ML circumvents the challenges posed by environmental and device variation to provide robust quantitative analysis.^81^ Similarly, de Almeida et al. performed a COVID-19 saliva-based screening using MALDI­(+) FT-ICR mass spectrometry-based SVM classifiers, wherein optimized trypsin digestion and spectral modeling yielded 95.6–100% accuracy and 0% false negatives, demonstrating the diagnostic capability of mass spectrometry-ML integration in rapid screening during pandemics (Figure 3D).^82^ Additionally, Martinez-Cuazitl et al. used ATR-FTIR spectroscopy followed by multivariate linear regression to identify unique vibrational “fingerprints” in the saliva of 255 patients infected with COVID-19 compared to healthy controls. The study discriminated clearly between groups based on amide and immunoglobulin spectral shifts, producing a noninvasive, low-cost alternative diagnostic route.^83^ Stepping up from saliva to nasopharyngeal swabs, work explored a smartphone-imaged electrochemiluminescence (ECL) immunoassay analyzed via an ANN (see Figure 3E). Trained on 45 samples and tested on 50 unknown swabs, a diagnostic accuracy of >90% and *R* ^2^ ≈ 0.94–0.95 in prediction of RT-PCR Ct values (up to 32 cycles), while detection of viral antigens down to 10^–12^ g/mL establishes a potentially robust, portable and quantitative diagnostic assay beyond the clinical standard.^84^ In combination with this, another work used ATR-FTIR spectroscopy combined with PLS-DA classification on 1,116 clinical swabs (558 PCR-positive/558 negative). Using PCR as the reference approach, the model had an AUROC ≈ of 0.68, an accuracy ≈ of 0.63, specificity ≈ of 0.64, with performance limited by dilution in transport medium, suggesting that swab analysis could provide significant diagnostic improvement, as shown in Figure 3F.^85^ Collectively, these studies demonstrate a smooth technological interplay of spectrum techniques such as spectroscopy, impedance sensing and colorimetry with fluorescence and electrochemiluminescence methods, suggesting that AI/ML methods can vastly improve accuracy metrics and reduce reliance on trained users while simultaneously decreasing turnaround time. Importantly, these matrix values, high accuracy, AUROC, sensitivity, and ultralow LODs, indicate analytical performances comparable to, or better than, conventional lab-based RT-PCR or ELISA systems, yet with faster processing and minimal invasiveness. In terms of technical readiness, these AI–biofluidic platforms are largely at TRL 5–7, indicating validation in relevant or limited clinical settings but still requiring multicentric trials, standardization of data pipelines, and regulatory integration. The future path involves aligning data sets, integrating explainable AI to enhance model transparency, and constructing affordable and disposable chips for scalable deployment. As these technologies develop, saliva- and swab-based AI diagnostics will revolutionize precision healthcare, moving diagnostics from centralized laboratories to smart, connected, patient-completed point of care ecosystems.

### 8.2 AI-Integrated Sweat Biosensors for Continuous and Personalized Health Monitoring

AI-Assisted sweat-based disease diagnosis is an innovative approach that utilizes AI/ML algorithms in conjunction with wearable sweat biosensors for the diagnosis of diseases through the analysis of sweat. It allows for the continuous collection and analysis of various biochemical and physiological markers like glucose, cortisol, lactate, and electrolytes, thus enabling real-time monitoring of patients’ health conditions.^86^ This fusion enables rapid, personalized, and continuous disease assessment without the need for invasive blood sampling or sophisticated laboratory infrastructure, paving the way for intelligent, patient-centric clinical decision-making.^87^

Beginning with the idea of monitoring cortisol, Shahub et al. developed a flexible nanoporous electrochemical sensor for sensing cortisol (8–140 ng/mL) in passive sweat (Figure 4A). Electrochemical Impedance Spectroscopy (EIS) data were processed with a weighted *k*-NN algorithm, classifying rising and falling cortisol trends with 100% accuracy and high repeatability (∼<20%). Validated by k-means cross-validation, the model produced a 100% true-positive rate and 0% false-negative rate, an on-demand circadian rhythm tracker 6 to 10 times cheaper and more comfortable than blood or saliva assays.^88^ Building on this, Liu et al. presented a configurable colorimetric chip (Figure 4B) consisting of sodium alginate gel capsules containing enzymatic indicators for glucose, pH, and lactate detection. Models were trained on 4600 colorimetric images, where CNN achieved 100% classification accuracy, and 91–99.7% correlation with lab spectrometry”.^89^ Through Class Activation Mapping, the model’s interpretability improved, making this reagent-efficient, instrument-free system faster and more portable than traditional HPLC or EIS assays. Extending the concept, Zhang et al.^90^ developed a self-calibrating hydrogel-based colorimetric patch for in situ sweat analysis of Zn^2+^, glucose, and Ca^2+^. A CNN trained on 5625 images achieved 100% accuracy and 91.7–97.2% agreement with UV–Vis data (see Figure 4C). It is absorbing–swelling hydrogel automatically compensated for sweat volume, removing calibration errors and enabling low-cost, power-free, real-time sensing, a major advance in wearable diagnostics.

**Figure 4:**
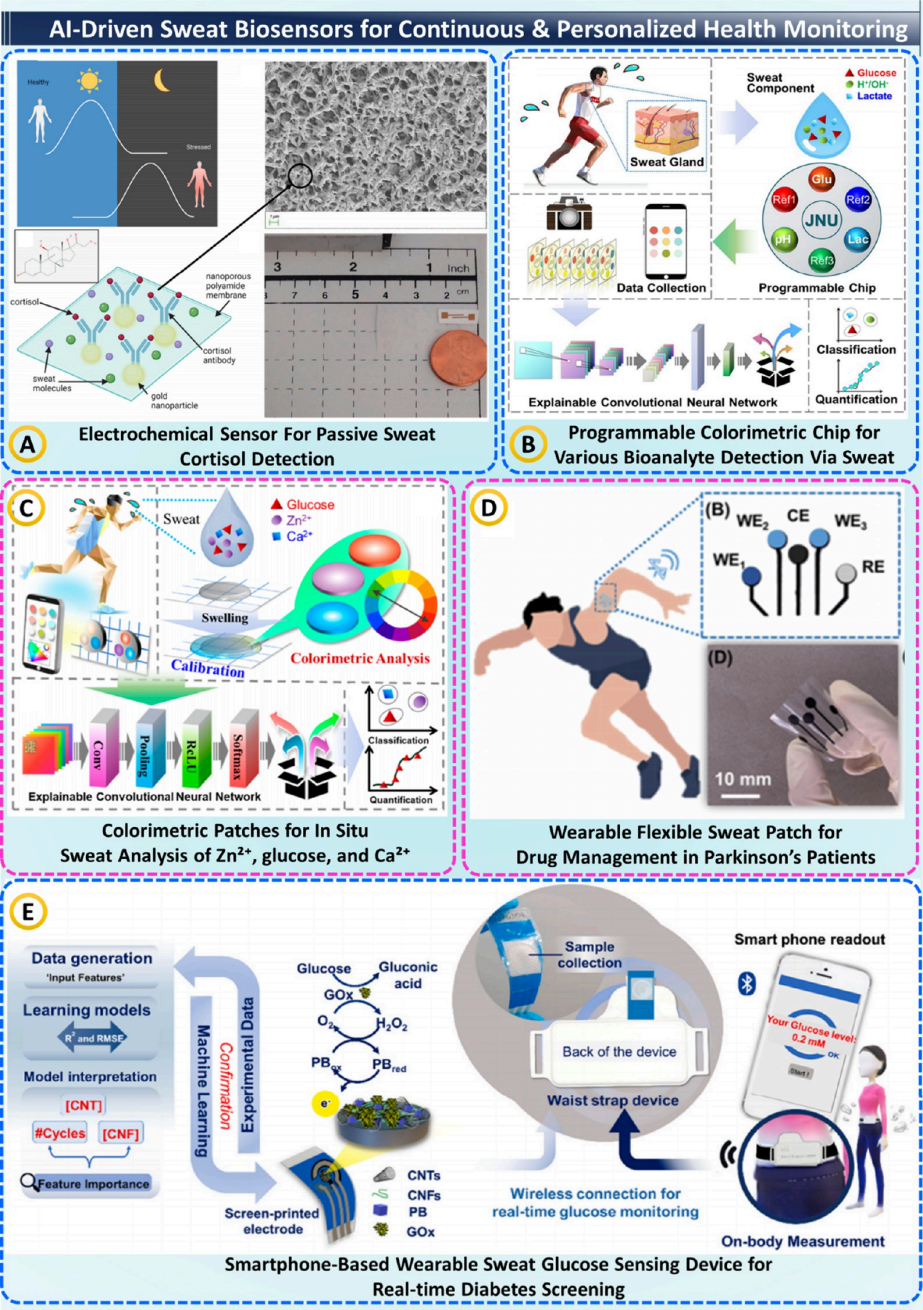
AI-driven sweat biosensors for continuous, personalized monitoring. (A) Passive electrochemical cortisol sensor (nanoporous polyimide/Au NP), taken from^88^ with the permission of Elsevier. (B) Programmable colorimetric microfluidic chip + explainable CNN for multianalyte (glucose/lactate/pH/ions) readout, taken from^89^ with the permission of ACS. (C) On-skin colorimetric patches for Zn^2+^, glucose, and Ca^2+^ with smartphone-CNN calibration, taken from^90^ with the permission of ACS. (D) Flexible wireless sweat patch (multielectrode array) for drug management in Parkinson’s, taken from^91^ with the permission of ACS. (E) Smartphone-connected sweat-glucose device (SPCE + GOx) with ML analytics for real-time on-body readout, taken from^92^ with the permission of Elsevier.

Transitioning to electrochemical platforms, Promphet et al.^92^ created a smartphone-linked glucose body strap featuring a CNTs–CNFs–Prussian blue nanocomposite. ML regression correlated electrochemical current with material content and deposition cycles, yielding a 0.1–1.5 mM linear range, LOD 0.1 mM, and 0.3 mM cutoff for diabetes screening. The Bluetooth-connected system provided real-time, painless glucose monitoring (see Figure 4E), outperforming finger-prick tests with ML-guided optimization. Expanding to neurodegenerative care, Yu and Tang^91^ introduced an ANN-assisted g-C_3_N_4_-based sweat patch for L-Dopa and glucose tracking in Parkinson’s patients (see Figure 4D). Using multimodal data sets (pH, time, activity), ANN achieved the best predictive accuracy for personalized dosing. It is an enzyme-free, stable nanomaterial and IoT integration enabled adaptive, real-time drug management, surpassing conventional enzyme-dependent methods. Complementing these, Yüzer et al.^93^ designed “DeepLactate,” a smartphone-embedded μPAD with Inception-v3 CNN for lactate detection. Trained on multidevice images, it achieved 99.9% accuracy with <1s processing and worked offline, monitoring postexercise lactate without cloud dependence. Finally, Bao et al. introduced an ML-aided CB–GO/CP flexible electrode for the detection of tyrosine in sweat and urine. Using ANN and SVM trained models, and taking inputs of pH and temperature, high-accuracy multivariate predictions of amino acid biomarkers were achieved, thereby allowing quicker, low-cost, portable analysis in contrast to resource-hungry chromatographic methods.^94^ Together, such trends communicate a message of high analytical accuracy (91–100%) and reproducibility (≤20% variability) of relevant clinical biomarkers in sweat, rapid turn-around-times, and enhanced patient engagement and access, compared to current diagnostic approaches. With a TRL of 5–7, further lab-to-field validation in home-based healthcare monitoring by lay users is attractive and promising; additionally, larger studies on validation, standardization, and drift over time would ensure that these platforms reach TRL 8–9 for inclusion in mainstream, subscription-based, personalized, continuous clinical diagnostics.

### 8.3 AI-Driven Urine Analytics for Cancer Detection and Organ Function Evaluation

Urine, an easy-to-collect biofluid, is a treasure trove of biomarkers, proteins, metabolites, nucleic acids, and extracellular vesicles that convey information on global physiology and pathology. For various types of urine signals derived from sensors, spectral and optical signals can be translated with great fidelity into diagnostic information with AI/ML-inspired data analytics. With this new paradigm, responsive diagnostic information could be produced in noninvasive, rapid and scalable forms, in contrast to the traditional test laboratory, which is too often invasive, slow and expensive.^95–97^

Urine diagnostics integrated with AI/ML recently achieved disease detection through intelligent data processing and nanostructured architectures. The work of Vo Thi et al. developed a 3D plasmonic coral nanoarchitecture coupled with a hand-held Raman reader, contrasting prostate and pancreatic cancers with deep learning models directly from urine, as demonstrated in Figure 5A. This label-free SERS platform achieved high sensitivity and specificity as a rapid, noninvasive alternative to chromatographic and ELISA-based analyses, which require reagents and complex instrumentation.^98^ Extending this approach, Muhammad Shalahuddin et al. used a 96-well 3D gold nanoarchitecture (Figure 5B), capable of high-throughput SERS of whole urine. ML correctly classified five cancer types with 95.6% accuracy, LOD of 1.23 × 10^–9^ M and reproducibility within ∼ 10% RSD, revealing potential for a scalable and rapid platform. This new strategy provides a significant advantage over LC–MS and NMR panels, which while accurate, are lengthy and expensive.^99^ Extending the diagnostic spectrum from cancer to infectious diseases, Jianyu Yang et al. developed a colorimetric Fe single-atom nanozyme array integrated with ML algorithms to identify urinary tract infection pathogens, as shown in Figure 5C.

**Figure 5:**
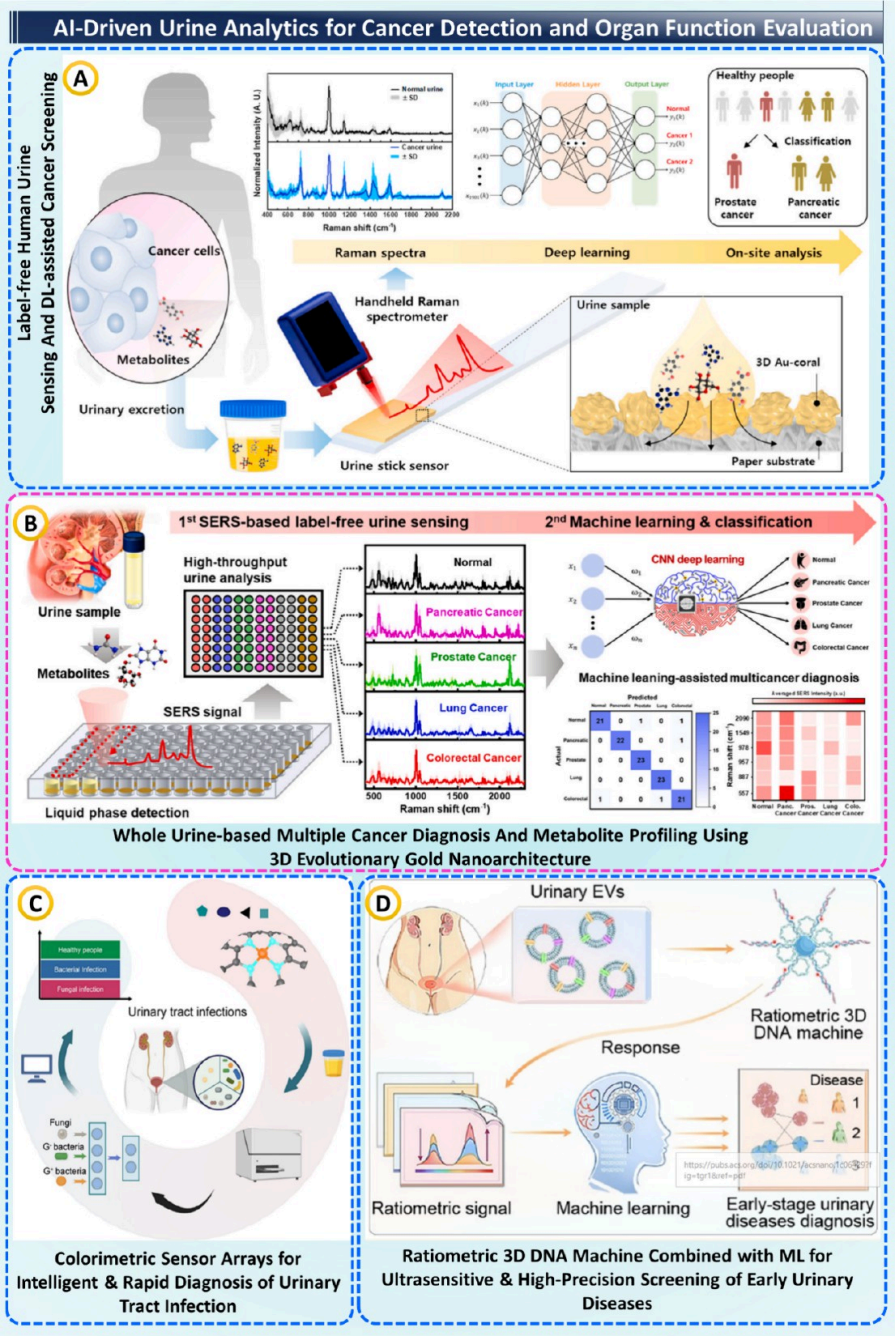
AI-driven urine analytics for cancer detection and organ function. (A) Label-free urine SERS + hand-held reader with DL for on-site cancer screening, taken from^98^ with the permission of Elsevier. (B) High-throughput 3D-Au SERS platform with ML/CNN classification of multiple cancers, taken from^99^ with the permission of Elsevier. (C) Colorimetric sensor array with intelligent analysis for rapid UTI diagnosis, taken from^100^ with the permission of ACS. (D) Ratiometric 3D-DNA machine + ML using urinary EVs for ultrasensitive early stage disease screening, taken from^101^ with the permission of ACS.

The system achieved up to 97% accuracy in analyzing 60 clinical urine samples within just 1 h, marking a dramatic improvement over conventional urine culture and flow cytometry, both of which require longer incubation and costly reagents.^100^ Moving to molecular-level biomarker profiling, Na Wu et al. proposed a radiometric 3D DNA machine for the detection of urinary extracellular vesicles for early identification of renal and urological diseases (see Figure 5D). Using SVM and KNN algorithms, it attained 95–100% classification accuracy with 40 patient samples, outperforming invasive cystoscopy and low-sensitivity cytological methods.^101^ Further extending this concept, Na Wu et al. developed a multimodal ML fusion approach that combined fluorescence, ICP-MS, and UV–vis signals to diagnose bladder cancer. This approach offered much better reliability compared to single-modality tests with 95% accuracy, 93% precision, and 93% recall, thereby underlining the importance of data fusion for enhancing predictive robustness and clinical precision.^102^ Broadening the applicative scope beyond cancer, Yidan Wang presented a three-in-one multifunctional nanoparticle array that can qualitatively, quantitatively, or classifying analyze, classify five proteinuria types. Moreover, producing an AUC ≈ of 0.975 in 10 min, the platform grouped all aforementioned urine tests into one fast assay, allowing clinical practitioners to spend less time on diagnosing kidney disease, therefore potentially making it more available for decentralized diagnostics.^103^

Extending this noninvasive paradigm to organ transplant management, Xi Chen et al.^104^ utilized DL-enabled SERS mapping to distinguish between kidney allograft injuries such as delayed graft function, calcineurin toxicity, and immune-mediated rejection. With an overall accuracy of 93.03%, this method offered a safer, faster alternative to biopsy, supporting early intervention and long-term graft monitoring. Finally, bridging into oncological metabolite detection, M. Hassani-Marand^105^ designed an AI-assisted multicolorimetric assay using gold nanorods with silver metallization to detect neuroblastoma biomarkers, homovanillic acid and vanillylmandelic acid, simultaneously. The model achieved 100% sensitivity and specificity (*R* ^2^ > 0.99), effectively eliminating the dependence on ELISA or LC–MS methods.

The performance metrics, both analytical and clinical, of urine diagnostic studies with AI/ML components illustrate, across the various case studies, a substantial step increase in performance metrics such as sensitivity and specificity, accuracy ratings of 93–100%, nanomolar–picomolar LODs, and turnaround time with reports consistently less than 1 h. As previously stated, the precision and reproducibility of these forms of approach to urine diagnostics ≤ 10% RSD, predict strong advancements toward a TRL rating of 6–8, compete for clinical validations and real-world implementation, and the results approach expected clinical thresholds for use. The supremacy of the AI/ML platforms over conventional methods, as stated across the studies, reinforces their potential to provide automated, real-time, and reagent-free diagnostics toward personalized, point-of-care, and data-driven healthcare ecosystems. Future large-scale multicentric trials, regulatory harmonization, and integration with cloud-based AI analytics would thus be required to enable completely automated noninvasive urine diagnostics for early disease screening and personalized monitoring.

### 8.4 AI-Enabled Tear Biomarker Sensing for Ocular and Systemic Disease Diagnosis

In recent years, AI/ML have transformed tear-based diagnostics into a noninvasive, intelligent clinical decision framework that offers real-time insight into ocular and systemic health. Tears are a repository of biomarkers, including proteins, electrolytes, metabolites, and nucleic acids, reflecting diseases of the ocular surface, metabolic disorders, and endocrine disorders. Researchers have combined DL algorithms with wearable microfluidic, electrochemical, and colorimetric sensors; thus, achieving unprecedented precision, enabling quantitative health monitoring with no invasive blood sampling.

A pioneering example is the AI-assisted wearable microfluidic colorimetric system developed by Zihu Wang et al.,^106^ this platform used a PDMS-based flexible patch for simultaneous vitamin C, pH, Ca^2+^ and proteins in tears (Figure 6A). A cloud-connected CNN-GRU model corrected for signal distortions caused by variable lighting and pH variations, achieving *R* ^2^=0.998 for pH (others *R* ^2^=0.994). The platform analyzed tears in 20 μL in seconds, much faster and more convenient than benchtop spectrometric assays, demonstrating high TRL 7 for personal telehealth. Similarly, Surachate Kalasin et al.^107^ developed a lab-on-eyeglasses platform using a copper-containing benzenedicarboxylate (Cu-BDC) graphene oxide hybrid MOF electrode for detecting tear creatinine, a proxy for renal function. The electrochemical impedance spectroscopy-based device attained 95.1% selectivity and 83.3% ML-based predictive accuracy for estimating serum creatinine levels, as shown in Figure 6B. These wearable monitors provide real-time, continuous kidney status at the point-of-care, which has applications for care of the vulnerable and for use in telemedicine. In their work on developing shed biomarkers of disease, Xingyi Shu et al. bioinformatics and ML-based screening to characterize the proteomes of tears in relation to thyroid eye disease. Using multiple GEO data sets and weighted gene coexpression network analysis, they identified 84 lacrimal-associated genes and their candidate genes, or KIAA0319 and PRDX4, for a putative diagnostic biomarker. The resulting ML-based nomogram accurately discriminated TED-associated lacrimal dysfunction, emphasizing the role of AI in precision ophthalmic genomics.^109^ A study by Andrea Storås et al. used explainable ML (LGBMClassifier + SHAP) on proteomic tear data from 234 patients to identify proteins associated with meibomian gland dysfunction severity. The model identified potentially significant protein features associated with gland dropout and lipid-layer stability, offering transparency with diagnostic accuracy, which is crucial in fostering clinical trust in AI-based ophthalmic diagnostics.^110^

**Figure 6:**
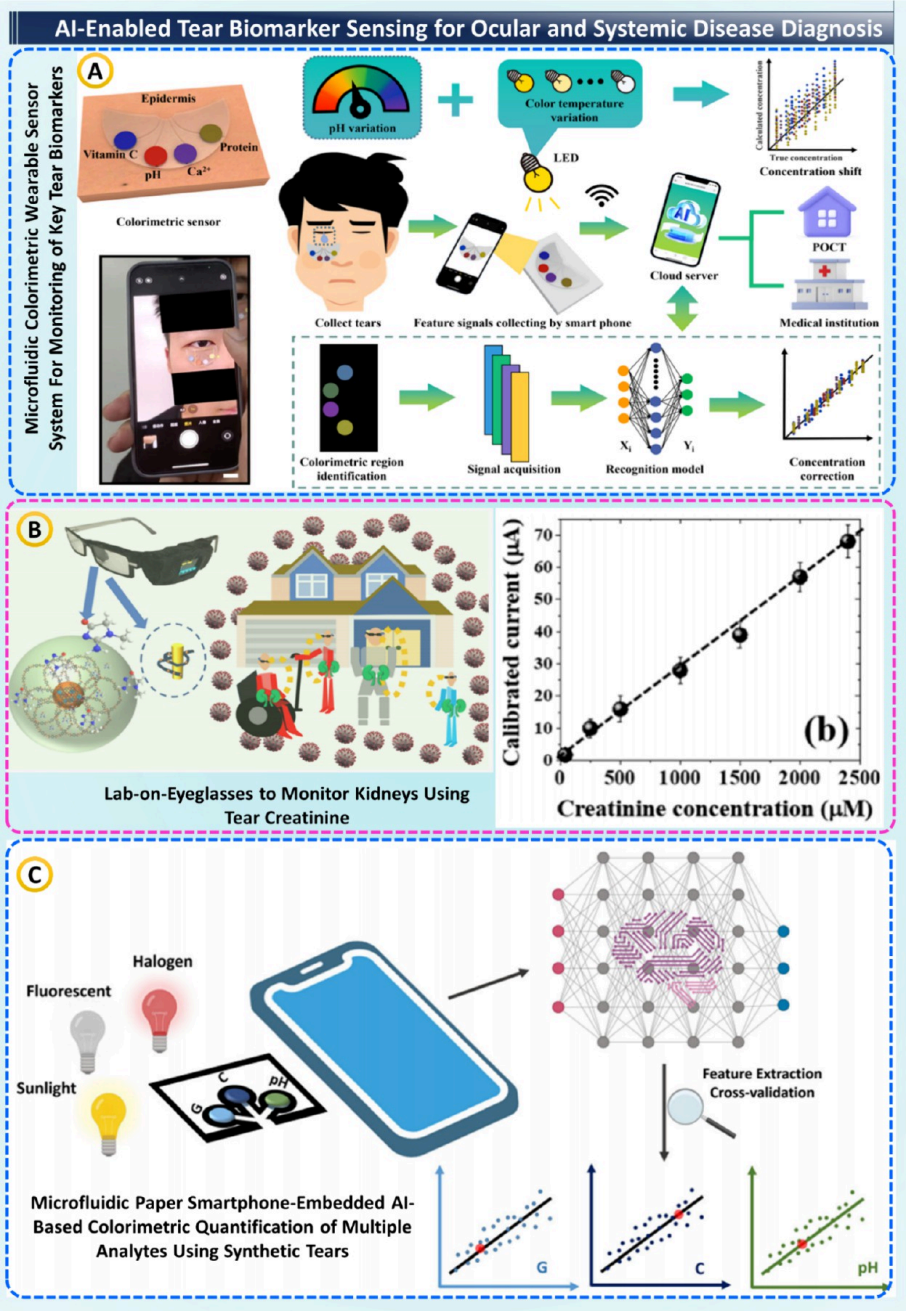
AI-enabled tear biomarker sensing for ocular/systemic diagnostics. (A) Microfluidic colorimetric wearable + smartphone/AI pipeline correcting illumination to quantify tear pH, proteins, vitamins, and ions, taken from^106^ with the permission of Springer. (B) Lab-on-eyeglasses electrochemical platform measuring tear creatinine for kidney monitoring, taken from^107^ with the permission of ACS. (C) Paper-microfluidic, phone-embedded AI quantification of multiple analytes in synthetic tears under varied lighting, taken from^108^ with the permission of Wiley.

To advance practical translation, Meliha Bastürk et al. developed a smartphone-embedded AI regression model (ChemiCheck app) in combination with a microfluidic paper-based device (μPAD) for synthetic tear analysis. The system was capable of quantifying glucose, cholesterol, and pH with limits of detection at 131 and 217 μM, and RMSE = 0.386. The app was able to make real-time inference in under one second without an Internet connection - an imperative step toward point-of-care deployment in resource-limited settings (Figure 6C).^108^ In summary, the studies collectively demonstrate an effectiveness that is equal to or better than laboratory-based methods, with 93–100% accuracy, submillimolar LODs, and ≤10% relative RSD precision, while also reducing the analytical time and level of invasiveness compared to laboratory approaches. TRL has been indicated as TRL 6–8, indicating that tear diagnostics can soon be used in clinical practice, and in the future should focus on cross-cohort validation, regulatory harmonization, and being incorporated using IoT technologies as AI models are developed toward multimodal data sets (spectral + image + genomic) to take these high precision tear diagnostic systems into clinically approved, patient-facing decision support tools for ocular and systemic disease management.

## 9 Challenges and Possible Solutions

However, clever and insight-generating these analyses and platforms may be, our desire and ability to use them is unfortunately fetishistically inhibited by important scientific, technical, and regulatory barriers to large-scale adoption. Moving from the controlled environment of the lab to the myriad of challenges associated with real-world use will definitely throw up challenges beyond those above, including limited and biased data, glitches in signal and domain drift across device or site, and lack of multicentric appeals validation. In addition to barriers arising from the difference in environments, edge-level deployment also creates potential constraints around computing capacity, as well as data security and privacy, in addition to the critical regulatory and interoperability considerations that are necessary for clinical adoption. Addressing these challenges necessitates a coordinated approach that encourages strong data governance, adaptive learning algorithms and practices, standardized validation methods, and secure federated infrastructure. The following sections drill deeper into each of the major roadblocks, which include data and labeling, domain drift, generalization and validation, edge constraints, and regulatory integrations, and provide tangible, practical, and technical pathways to support the transfer from potential prototypes to practical, resilient, and scalable clinical decision-making tools in daily life.

### 9.1 Data Scarcity and Labeling Quality in Biofluidic AI Systems

In AI-biofluidic systems, data and corresponding labeling present numerous complexities, particularly in experimental settings reliant on smaller, imbalanced, and noisy data sets (with small sample sizes). There will usually be only a small number of patient samples available for multiomic research (as would be the case for most studies). When patient samples are used for analysis purposes, most studies have too few positive to negative patient samples ratios to establish any statistical accuracy in the training of models.^111^ Ground truth labels are also not certain, as they depend on subjective interpretations by clinicians or imperfect reference assays or protocols. Further, batch effects due to differences during collection, storage, or use of a sensor lot introduce nonbiological variability. For these difficulties, researchers might enlist self-or supervised learning to leverage unlabeled data, use synthetic data augmentations to create variability from intersample diversity, and implement stability selection to capitalize on robust features. Finally, cross-site or interlaboratory harmonization of protocols can ameliorate some of the batch variability, improve models’ generalizability, and enhance reproducibility.^112,113^

### 9.2 Domain Shift, Signal Drift, and Model Adaptability

Domain shift and drift refer to performance degradation due to differences in the data distribution at deployment from the one used during training. It commonly arises in AI–biofluidic systems because of lot-to-lot variations in electrodes or reagents, differences in user handling, and changes in the biofluid matrix (e.g., pH, viscosity, or ion content changes).^114^ Such inconsistencies change sensor signals in ways that can confuse an AI model expecting consistent patterns. For domain adaptation, techniques such as transfer learning or feature alignment are used to re/target a given model to a different device or site. Periodic recalibration is performed to maintain performance due to changes in conditions. Drift monitoring continuously tracks deviations in predictions, while guardrails compare old versus updated models to ensure reliability and regulatory safety during field deployment.^115,116^

### 9.3 Validation, Generalization, and Clinical Reproducibility

Validation and generalization remain important bottlenecks to the translation of AI–biofluidic models to clinical use. Many systems have issues with overfitting, since they may perform well only on the site, device, or data set they were trained on, due to the data usually being very limited and homogeneous. A lack of prospective multisite clinical trials further diminishes confidence in broad applicability.^117,118^ External validation on independent data sets or with other cohorts of hospitals is, therefore, required to prove robustness. Analysis protocols are preregistered to avoid selective reporting and ensure reproducibility. Lastly, adherence to guidelines on TRIPOD-AI ensures standardized documentation of data set sources, preprocessing, and model performance for clarity, comparability, and building trust with clinicians.^119,120^

### 9.4 Edge-Level Constraints, Privacy, and Cybersecurity

However, edge constraints and security are significant deployment challenges for real-world, portable, or wearable AI–biofluidic systems. Applications deployed at the edge-for example, in smartphones, microfluidic readers, or patches-suffer from limited power, memory, and computational resources, which inherently limit complex AI inference.^121–123^ Simultaneously, concerns about data privacy and cybersecurity arise when sensitive health data are transmitted or stored outside of the device, including vulnerability to adversarial or spoofing attacks that can manipulate sensor signals or model predictions. In response, ML enables lightweight, energy-efficient AI models optimized for local computation. Federated analytics enables collaborative model training on-device without the need to share raw data, and secure aggregation enables the exchange of encrypted model parameters. Finally, continuous on-device quality control monitors signal integrity and detects anomalies, ensuring reliability in the data measured and the safety of the patient.^124^

### 9.5 Regulatory Integration and System-Level Interoperability

Regulatory and integration issues are the prime determinants for whether AI–biofluidic systems can make a leap from prototype to trusted clinical tools. Meeting compliance involves both analytical and clinical validations according to standards such as CLIA, FDA, and CE, ensuring sensor accuracy, reproducibility, and safety in real-world conditions.^125^ Furthermore, the categorization of a companion app is significant, as mobile or cloud-based interfaces often constitute medical software and have regulatory standards to meet. Periodic postmarket surveillance will monitor the performance of a device and model drift in regards to patient safety postdeployment. EHR and HL7-FHIR integration encourages seamless adoption in healthcare by facilitating interoperability to a clinical data system that allows automated updates and clinician access to patient population data.^126,127^ A condensed roadmap summarizing (Table 2) short-term (0–24 months) and long-term (3–5 years) strategies for AI–enabled biofluidic system deployment. It highlights essential objectives, key actions, and the coordinated roles of major stakeholders.

**Table 2:** Concise AI–Biofluidics Action Plan (Short-Term: 2 Years | Long-Term: 5 Years)

**time horizon**	**objective**	**key focus areas**	**lead stakeholders**	**expected outcomes**
0.5 years	set up governance and pilot scope	Steering committee, data governance, use-case selection (POCT, TDM, monitoring)	agencies, hospitals, universities	defined roadmap, IRB-ready protocols
0.5–12 years	pilot data generation	single-site pilots, model baselines, training/validation data sets	hospitals, researchers	validated models, data set
1–2 years	multisite validation and regulatory prep	cross-site calibration, external validation, TRIPOD-AI reporting	agencies, hospitals	submission-ready evidence-based
3 years	regulatory clearance and deployment	FDA/CE/CDSCO submissions, PMS setup, 10–20 site rollout	agencies, industry	market-ready product, postmarket safety monitoring
year 4	portfolio expansion	add new assays, localization, and interoperability upgrades	universities, industry	expanded test menu, multicountry compliance
year 5	sustainable & learning system	federated continual learning, value-based care, sustainability & EPR	researchers, hospitals, policy bodies	self-learning network, reduced cost and carbon impact

Taken together, these challenges and their solutions, as presented in Section 5, suggest that biofluidics AI development needs less technology and more system coordination. It outlines a genuine path forward for how to tackle the data challenge, domain drift, regulation and bottlenecks to deployment over both the short and longer time scales. This roadmap provides an important bridge toward Section 6, to extend out of the nitty-gritty operational needs into the skyward vision of where AI-capable biofluidics systems can take us in creating holistic, scalable, interoperable, and globally distributed diagnostics systems.

## 10 Conclusion and Future Scope

The intersection of biofluidics and AI represents a new paradigm in diagnostics by converting small volumes of biological fluids into immediate clinical insights that can be acted upon. The combination of the precision of microfluidics with the intelligence provided by AI/ML algorithms enables the barriers of laboratory testing to be bypassed. When that type of functionality is made into a small, portable device, it helps mitigate costs and provides a minimally invasive modality to the issue of personalized health and continuous monitoring. Platforms built on saliva, sweat, urine, and tears, for example, each make use of AI to improve the sensitivity, specificity, and reproducibility of testing. AI will also be invoked in the URL for dynamic calibration, drift correction, and explainable AI.

While we have made significant strides, translation from laboratory prototypes into regulated clinical trials is hindered by data limitations, bioliquid matrix variability, and lack of multicentric validation. Harmonized data sets, analytical-ML benchmarking, and clear reporting frameworks are imperative. It is then embedding federated and continual learning architectures that will enhance generalizability while ensuring data sanitization and privacy.

In the near term, such collaborative networks involving hospitals and regulators, along with academic centers, should focus on large-scale real-world trials (TRL 6–8) in order to establish analytical credibility and clinical utility. In the longer term, integration with EHR and digital twins will yield predictive, longitudinal, and adaptive health ecosystems. Some of the burgeoning growth areas include hybrid biofluidic-wearable systems for multianalyte sensing, edge deployable AI/ML for autonomous inference and explainable AI pipelines for regulatory compliance. The synergy of AI and biofluidics has the potential to democratize diagnostics, bestowing lab-grade intelligence upon every drop of biological fluid and every point of care. This evolution will arm clinicians with quick, data-driven decisions, while also placing patients at the heart of a smart, connected, and proactive healthcare system.

## Data Availability

Data is included within the manuscript.
